# Safety and Immunogenicity of Chimeric *Pestivirus* KD26_E2LOM in Piglets and Calves

**DOI:** 10.3390/vaccines11101622

**Published:** 2023-10-21

**Authors:** Gyu-Nam Park, Jihye Shin, SeEun Choe, Ki-Sun Kim, Jae-Jo Kim, Seong-In Lim, Byung-Hyun An, Bang-Hun Hyun, Dong-Jun An

**Affiliations:** 1 Virus Disease Division, Animal and Plant Quarantine Agency, Gimcheon 39660, Republic of Korea; changep0418@korea.kr (G.-N.P.); shinji227@korea.kr (J.S.); ivvi59@korea.kr (S.C.); kisunkim@korea.kr (K.-S.K.); jkim1209@korea.kr (J.-J.K.); saint78@korea.kr (S.-I.L.); hyunbh@korea.kr (B.-H.H.); 2 College of Veterinary Medicine, Seoul University, Gwanak-ro, Gwanak-gu, Seoul 08826, Republic of Korea; anbh5043@gmail.com

**Keywords:** BVDV, CSFV, chimeric, pig, calf, antibody

## Abstract

A chimeric *pestivirus* (KD26_E2LOM) was prepared by inserting the E2 gene of the classical swine fever virus (CSFV) LOM strain into the backbone of the bovine viral diarrhea virus (BVDV) KD26 strain. KD26_E2LOM was obtained by transfecting the cDNA pACKD26_E2LOM into PK-15 cells. KD26_E2LOM chimeric *pestivirus* proliferated to titers of 10^6.5^ TCID_50_/mL and 10^8.0^ TCID_50_/mL at 96 h post-inoculation into PK-15 cells or MDBK cells, respectively. It also reacted with antibodies specific for CSFV E2 and BVDV E^rns^, but not with an anti-BVDV E2 antibody. Piglets (55–60 days old) inoculated with a high dose (10^7.0^ TCID_50_/mL) of KD26_E2LOM produced high levels of CSFV E2 antibodies. In addition, no co-habiting pigs were infected with KD26_E2LOM; however, some inoculated pigs excreted the virus, and the virus was detected in some organs. When pregnant sows were inoculated during the first trimester (55–60 days) with a high dose (10^7.0^ TCID_50_/mL) of KD26_E2LOM, anti-CSFV E2 antibodies were produced at high levels; chimeric *pestivirus* was detected in one fetus and in the ileum of one sow. When 5-day-old calves that did not consume colostrum received a high dose (10^7.0^ TCID_50_/mL) of KD26_E2LOM, one calf secreted the virus in both feces and nasal fluid on Day 2. A high dose of KD26_E2LOM does not induce specific clinical signs in most animals, does not spread from animal to animal, and generates CSFV E2 antibodies with DVIA functions. Therefore, chimeric *pestivirus* KD26_E2LOM is a potential CSFV live marker vaccine.

## 1. Introduction

Bovine viral diarrhea virus (BVDV, *pestivirus bovis and pestivirus tauri*) and classical swine fever virus (CSFV, *pestivirus suis*) belong to the genus *pestivirus* within the family *Flaviviridae*; other members include border disease virus (BDV, *pestivirus ovis*) and several newly identified atypical *pestiviruses*. New *pestiviruses* include *Pestivirus antilocaprae*, *Pestivirus australiaense*, *Pestivirus aydinense*, *Pestivirus brazilense*, *Pestivirus ratti*, *Pestivirus scrofae*, *Pestivirus L*, *Pestivirus M*, *Pestivirus N*, *Pestivirus O*, *Pestivirus P*, *Pestivirus Q*, *Pestivirus R*, and *Pestivirus S*. These changes in pestivirus taxonomy have been approved and ratified by the International Committee on Taxonomy of Viruses (ICTV) in March 2022 (https://ictv.global/).

*Pestiviruses* have a positive single-stranded RNA genome of ~12.3 kb, which encodes a single open reading frame (ORF) that is translated into 12 viral polypeptides; the genome is flanked by untranslated regions (UTRs) at the 5′- and 3′-ends [1,2,3]. The ORF encodes a polyprotein of 3898 amino acids that, upon proteolytic processing, yields four structural proteins (Core, E^rns^, E1, E2) and eight nonstructural proteins (N^pro^, p7, NS2, NS3, NS4A, NS4B, NS5A, and NS5B) [1,2,4,5,6]. Pigs and wild boars infected with CSFV, a systemic disease with a very high fatality rate, show symptoms such as high fever and leukopenia [7,8]. CSFV infection is classified as acute, chronic, or persistent [7,8]. Acute infection is caused by a highly virulent strain, resulting in severe illness and high mortality within 2–4 weeks; however, pigs chronically infected with a moderately virulent strain survive for over 30 days [2]. Persistent infection (PI) is passed from sow to fetus during pregnancy and develops through immunotolerance mechanisms; the immature immune system of fetal and newborn pigs does not recognize CSFV [7,8].

BVDV, which has spread worldwide, is considered to be an important pathogen in cattle; the virus has an adverse economic impact, mainly through reproductive losses or birth of PI calves [9]. The main symptoms of BVDV depend on the virus genotype and biotype [9]. Pigs can sometimes be affected with this virus [10,11,12,13,14]. BVDV is classified into two genotypes, type 1 and type 2, which are sub-classified into BVDV-1 (1a to 1u; 21 sub-genotypes) and BVDV-2 (2a to 2d; four sub-genotypes) [3,15]. Based on their effects on the replication of cultured cells, BVDV isolates are characterized as CP or non-cytopathic (NCP), with the latter being responsible for most natural infections and PI of fetuses [16]. During the first trimester, infection of pregnant animals with NCP viruses of both species may cause fetal death or the birth of PI calves [17]. CP isolates, which constitute the minority, are isolated almost exclusively from cattle with mucosal disease [16]; however, the majority of BVDV infections in swine have no clinical signs [13,18].

Various studies have developed and tested vaccines to eradicate CSFV; however, most commercially available vaccines are live vaccines, and only an E2 Subunit vaccine and/or a chimeric *pestivirus* vaccine are used in some regions. Previous studies used a CSFV backbone or a BVDV backbone to develop a chimeric live *pestivirus* marker vaccine with DIVA (Differentiating Infected from Vaccinated Animals) function [19,20,21,22]. A BVDV/CSFV chimera, “CP7_E2alf”, carrying the CSFV E2 (Alfort 187 strain) and BVDV (CP7 strain) backbones maintained antibody titers for at least 6 months after a single intramuscular or oral vaccination and protected pigs against inoculation with virulent CSFV [19]. A BVDV/BDV chimera, “CP7_E2gif”, carrying the BDV E2 (Gifhorn strain) and BVDV (CP7 strain) backbones, confirmed the potential of the DIVA vaccine, with detection of anti-CSFV E2-specific antibodies after challenge with virulent CSFV [20]. A rC/bUTRs vaccine strain was also constructed by replacing the 5’- and 3’-UTRs, as well as partial E2 regions within the C-strain backbone, with equivalent genes from BVDV [21]. The Flc-LOM-BE^rns^ strain was constructed by replacing the partial 3′ end of the capsid gene and the complete E^rns^ gene with those of BVDV within the backbone of the CSFV (LOM strain) used as a CSF live attenuated vaccine [22]. Currently, only the Flc-LOM-BE^rns^ strain is used commercially as a live marker vaccine to eradicate CSF, and more than 5 million doses of this product are inoculated into Korean pigs every year. For effective CSF control, various live marker vaccines must be researched and commercialized continuously, which will help to eradicate CSF in countries around the world. In addition, recent studies inserted the E^rns^ gene of *pestiviruses* (Bungowannah virus, Norwegian mouse *pestivirus*, and Pronghorn *pestiviruses*), which do not share high homology, into infectious CSFV cDNA clones; this avoided using the E^rns^ genes of the main *pestiviruses* (A–D) [23,24,25]. The advantage of this strategy is that it ensures reliable differential detection of antibodies, making it a useful DIVA vaccine.

The purpose of this study was to develop a live marker vaccine in which the E2 gene was replaced by the CSFV E2 gene within the KD26 strain, which has the characteristics of the BVDV 1a genotype and CP type viruses, and to use this as a backbone. In addition, we conducted safety and immunogenicity tests for the KD26_E2LOM strain by inoculating growing pigs, pregnant sows, and calves with very high doses of the candidate vaccine.

## 2. Materials and Methods

### 2.1. pACKD26_E2LOM

**Figure 1:**
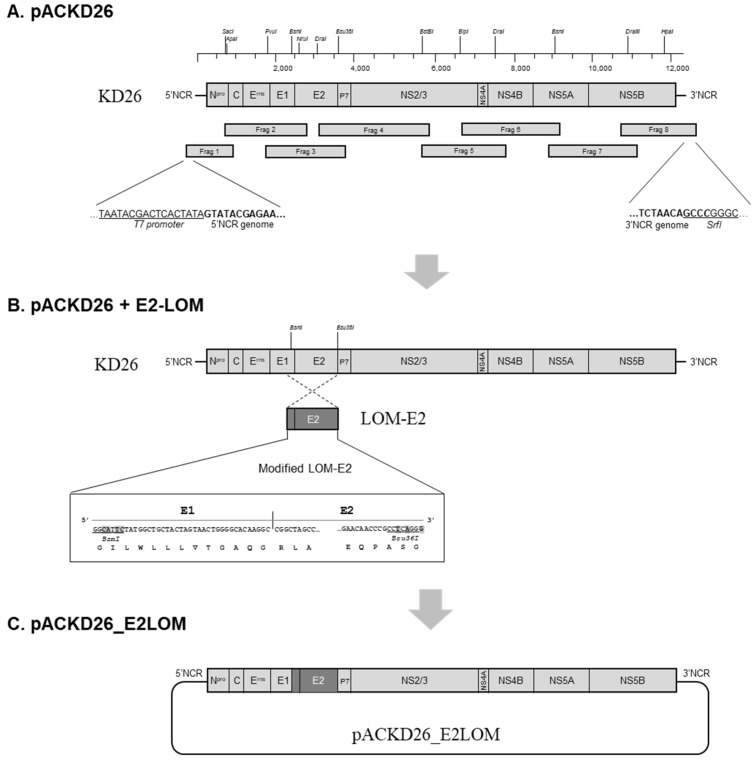
Schematic representation showing construction of the KD26_E2LOM infectious clone based on the BVDV1 KD26 genome. (**A**) Strategy for construction of a full-length BVDV1 KD26 cDNA clone (pACKD plasmid). Eight cDNA fragments encompassing the complete KD26 genome were amplified and assembled into the pACYC177 low-copy plasmid vector using restriction enzymes and DNA ligase. The T7 promoter and a *SrfI* site were added to the 5′-UTR and 3′-UTR of KD26 genome, respectively (underlined). (**B**) Strategy used to construct an infectious cDNA clone of KD26_E2LOM containing the LOM-E2-encoding region (pACKD26_E2LOM). The LOM-E2 cDNA fragment was modified by insertion of restriction enzyme sites (*BsmI* and *Bsu36I*) at either end to allow replacement of the KD26 genome. (**C**) Generation of pACKD26_E2LOM cDNA construct.

The infectious cDNA clone KD26_E2LOM was generated by replacing the E2 gene in non-cytopathic BVDV1 (strain KD26, genotype 1a, accession no. OR683432) with the E2 gene from CSFV (strain LOM) (Figure 1). First, a full-length cDNA clone of BVDV KD26 was constructed from eight cDNA fragments. Viral RNA from KD26 was extracted (QIAamp viral RNA kit; QIAGEN, Hilden, Germany) and cDNA synthesized using random hexamers (HelixCript™ Easy cDNA synthesis kit; NANOHELIX, Daejeon, Republic of Korea). Eight cDNA fragments covering the whole genome of KD26 were amplified by PCR (AccuPower^®^ Pfu PCR premix; BIONEER, Daejeon, Republic of Korea) using the eight primer sets listed in Table 1.

### 2.2. KD26_E2LOM Strain in Cell Lines

The KD26_E2LOM strain was maintained by passaging the virus in MDBK cells (ATCC number CCL-22). On the day before the virus infection, monolayers of MDBK cells were cultured to 80–90% confluency in α-minimum essential medium (α-MEM; GIBCO, Billings, MT, USA) supplemented with 5% horse serum (HS, heat-inactivated; GIBCO, USA), 2 mM L-glutamine (GIBCO, USA), 1 mM sodium pyruvate (GIBCO, USA), and 100 U penicillin/streptomycin (GIBCO, USA). After discarding the medium and washing the cells with phosphate-buffered saline (PBS), the recovered KD26_E2LOM virus was inoculated into fresh MDBK cells. The virus-inoculated MDBK cells were incubated at 37 °C/5% CO_2_ for 1 h. Thereafter, the virus inoculum was discarded and replaced with fresh growth medium. The virus-inoculated MDBK cells were incubated at 37 °C/5% CO_2_ incubator for 3 days, or until cytopathic effects were detected. Virus stocks were obtained by freeze-thawing the cultures three times and collecting the supernatant by centrifugation at 3720× *g* for 10 min. The supernatant was then passed through a syringe filter (0.45 μm) and used for the next passage.

### 2.3. Safety and Pig-to-Pig Transmission of KD26_E2LOM

Fifteen pigs aged 55–60 days, all of which were negative for CSF and BVD antibodies, were divided into three groups (Group 1, *n* = 7; Group 2, *n* = 4; and Group 3 (negative control), *n* = 4). Pigs in Group 1 were inoculated once with KD26_E2LOM (10^7.0^ TCID_50_/dose). Pigs in Group 2 were not inoculated but co-habited with pigs in Group 1. Blood and nasal samples were obtained for antibody titer and antigen tests during the experimental period (0, 1, 4, 7, 11, 14, 21, and 28 days of post-inoculation (dpi)). Each of the KD26_E2LOM-inoculated pigs and co-habiting pigs was autopsied on 7, 14, 21, and 28 dpi, respectively, and 11 organs (tonsil, heart, kidney, lung, liver, spleen, cecum, brain, ileum, inguinal lymph nodes, and mesenteric lymph nodes) were harvested to detect the presence of viral antigens by qRT-PCR.

### 2.4. Safety of KD26_E2LOM in Pregnant Sows

Three sows in the middle stages of pregnancy (55–60 days) were inoculated with KD26_E2LOM (10^7.0^ TCID_50_/dose) and observed until 21 dpi. Blood was collected on the day before (Day 0) and at 14 and 21 days after vaccination with KD26-E2LOM, and nasal swab samples were collected on 0, 4, 7, 14, and 21 dpi. Blood samples were investigated to detect changes in antibody levels. Blood, nasal, and fecal samples were also tested for KD26_E2LOM antigens. Pregnant sows were autopsied on Day 21, and 11 organs were harvested to detect KD26_E2LOM by qRT-PCR. The number of fetuses and the CR (crown-rump length [cm]) of pregnant sows was recorded, and qRT-PCR was used to confirm the presence of KD26_E2LOM in the fetuses.

### 2.5. Safety of KD26_E2LOM in Calves

Nine calves (5 days old) that did not consume colostrum were divided into three groups prior to the safety experiments. Group 1 (three calves) was inoculated with 10^7.0^ TCID_50_/dose KD26-E2LOM strain, Group 2 (three calves) with BVDV type 1 (BVD26 strain, 10^7.0^ TCID_50_/dose), and Group 3 (three calves) was used as a negative control. Blood, nasal, and fecal samples were collected before (−4 and 0 days) and after (2, 4, 7, 10, 14, and 21 days) inoculation with KD26-E2LOM or BVD26. Samples were also collected from calves in control Group 3.

### 2.6. Analysis of CSFV E2 and BVDV E2 by ELISA

Serological differential diagnosis in animals inoculated with the KD26_E2LOM vaccine strain is possible using the DIVA function, which determines CSFV E2 antibodies as positive and BVDV E2 antibodies as negative. Additionally, in the case of BVDV infection, the results were determined as BVDV E2 positive and CSFV E2 negative, and in the case of CSFV infection, the results were the opposite of those of BVDV infection. Serological ELISA analysis to determine specific antibody levels in serum was performed using commercially available kits. Levels of anti-CSFV E2 antibodies in pig serum were measured using the VDPro^®^ CSFV AB ELISA kit, which is an E2 protein-based ELISA (MEDIAN diagnostic Co., Cat No. ES-CSF-01, Chuncheon, Republic of Korea). The test serum samples were validated by comparing their OD values with those of the control (a mean OD value ≥ 0.5 was positive and a mean OD value < 0.3 was negative). Results were interpreted according to the sample to positive ratio (S/P ratio), calculated as follows: (mean OD value of sample—mean OD value of negative control)/(mean OD value of positive control—mean OD value of negative control). Test samples with an S/P ratio ≥ 0.14 are considered positive for CSFV E2-specific antibodies, and those with an S/P ratio < 0.14 are considered negative. ELISAs measuring antibodies specific for BVDV E2 in serum of pigs were performed using VDPro^®^ BVDV AB ELISA (MEDIANDiagnostic Co. Cat No. EB-BVD-01, Chuncheon, Republic of Korea) kits, respectively. The BVDV AB ELISA is a blocking ELISA designed to detect neutralizing BVDV E2 antibodies. Test samples were validated by comparison with the control OD values (mean OD value ≤ 0.2 as positive and ≥0.6 as negative). Interpretation involves calculating the S/P ratio as above. Samples with a value ≤ 0.7 were BVDV E2 positive and those with a value > 0.7 were negative.

### 2.7. qRT-PCR

Viral RNA was extracted from blood, organs, and swab samples using the RNeasy Mini kit (Qiagen Inc., Cat. No. 74104, Valencia, CA, USA) and used for quantitative real-time PCR (qRT-PCR) to measure the CSFV RNA copy number. The qRT-PCR analysis was performed using a VDx^®^ CSFV qRT-PCR kit (MEDIAN diagnostics Co., Cat. No. NS-CSF-31, Chuncheon, Republic of Korea) designed to detect the 5′-UTR of CSFV [22]. The PCR reaction was performed in a CFX Opus 96 Real-Time PCR System (Bio-Rad Laboratories, Inc., Cat. No. 12011319, Hercules, CA, USA) under the following conditions: cDNA synthesis (50 °C, 30 min) and initial inactivation (95 °C, 15 min), followed by 40 cycles of a two-step PCR comprising denaturation (95 °C, 10 s) and extension (60 °C, 60 s). The mean cycle threshold (Ct) values were calculated based on positive samples, which were considered positive if they had a threshold cycle number ≤ 40. The viral RNA copy number (genomic copies/mL) was calculated from standard curves showing a linear correlation between the logarithm of the copy number and the Ct value.

### 2.8. Serum-Neutralizing Antibody Assay

Anti-CSF-neutralizing antibodies were detected by a neutralizing peroxidase-linked assay (NPLA) according to the standards manual of the World Organization for Animal Health (WOAH) [26]. Heat-inactivated serum samples (56 °C, 30 min) were serially diluted 2-fold (1:2 to 1:2048) and neutralized by adding 200 TCID_50_ of the LOM virus for 1 h at 37 °C. The neutralized serum containing virus was inoculated into PK-15 cells (porcine kidney cells) and cultured for 3 days in a 37 °C incubator. The cells were then fixed in chilled 80% acetone and reacted with a commercial anti-LOM mAb (MEDIAN diagnostic Co., Cat No. 9013, Chuncheon, Republic of Korea). The cells were then stained using a VECTASTAIN^®^ ABC-HRP kit (Vector Laboratories Inc., Cat no. PK-4000, Newark, CA, USA) and an ImmPACT DAB Peroxidase (HRP) substrate (Vector Laboratories Inc., Cat no. PK-4100, USA). Neutralizing antibody titers were expressed as the reciprocal of the highest dilution that yielded 50% neutralization and determined to ≥10-fold positive antibodies according to the WOAH criteria.

### 2.9. Statistical Analysis

All statistical analyses were performed using GraphPad Prism 6. Statistical significance was evaluated by Student’s *t*-test, and a *p*-value < 0.05 was considered significant.

## 3. Results

### 3.1. Generation of an Infectious KD26-E2LOM cDNA Clone and Virus Recovery

Primers are designed to add the T7 promoter sequence to the 5′-UTR of the KD26 genome; this was performed to facilitate in vitro transcription. In addition, an *SrfI* recognition site was inserted at the end of the 3′-UTR to facilitate plasmid linearization (Table 1 and Figure 1). To assemble a complete full-length clone of KD26, all cDNA fragments were digested with the appropriate restriction sites and ligated into the pACYC177 low-copy plasmid (pACKD26 construct, Figure 1A). Next, a KD26_E2LOM cDNA construct was generated by substituting the E2-encoding region of KD26 with CSFV LOM-E2.

The LOM-E2 fragment, including a portion of E1 (40 bp at the end of LOM-E1), was amplified by PCR using LOM virus cDNA as a template. To replace the LOM-E2 encoding region with that of KD26, the LOM-E2 fragment was modified by inserting appropriate restriction sites (*BsmI* and *Bsu36I*) at either end of the E2 region using PCR (Table 1). The modified LOM-E2 was digested and ligated into a pACKD26 plasmid via the *BsmI* and *Bsu36I* sites, thereby generating a pACKD26_E2LOM cDNA construct (Figure 1B,C). The pACKD26_E2LOM cDNA plasmid was amplified in *Escherichia coli* DH5α competent cells (Enzynomics, Daejeon, Republic of Korea) and purified using an Exprep™ plasmid SV mini kit (GeneAll, Seoul, Korea). The sequence was confirmed by an ABI Prism 3730xl DNA sequencer (Cosmo Genetech Co., Seoul, Republic of Korea). Subsequently, the cDNA construct was linearized by digestion with *SrfI* and in vitro transcribed to viral RNA using a MEGAscript™ T7 transcription kit (Thermo Fisher, Waltham, MA, USA). The resulting in vitro-synthesized RNA was transfected into porcine kidney 15 (PK-15) cells and cultured for 3 days. The recovered KD26_E2LOM virus was selected by detecting positive cells by immunofluorescent LOM E2-specific mAbs. The virus was then serially passaged in PK-15 cells and Madin–Darby bovine kidney (MDBK) cells.

The genome of the rescued KD26_E2LOM virus, that of the KD26 strain originally used as a backbone, and that of the CSFV E2 gene, were compared and analyzed. There were changes in five nucleotides (nt: 770, 2423, 3495, 3981, and 8286); however, only four genes harbored amino acid changes in N^pro^, E2, NS2, and NS4B (Table 2).

### 3.2. Rescue of the KD26_E2LOM Strain

**Figure 2:**
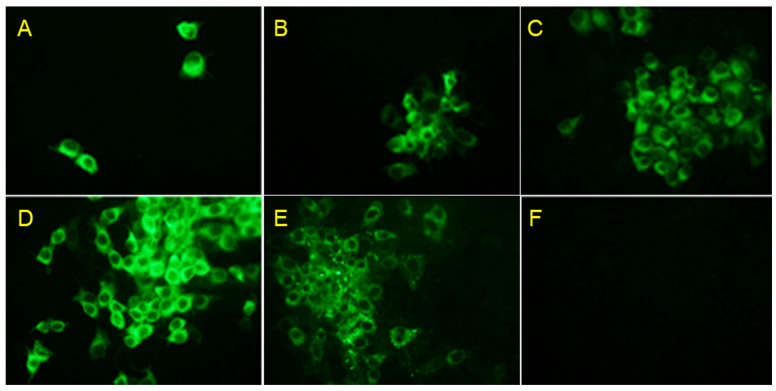
KD26_E2LOM virus was rescued after transfection of pACKD26_E2LOM. Indirect immunofluorescence analysis (IFA) using a CSFV E2-specific mAb was conducted at 24 h (**A**), 36 h (**B**), and 48 h (**C**) post-inoculation of KD26_E2LOM into PK-15 cells. IFA was also conducted using a CSFV E2-specific mAb (**D**), a BVDV Erns-specific mAb, and (**E**) a BVDV E2-specific mAb (**F**) after inoculation of KD26_E2LOM into PK-15 cells.

**Figure 3:**
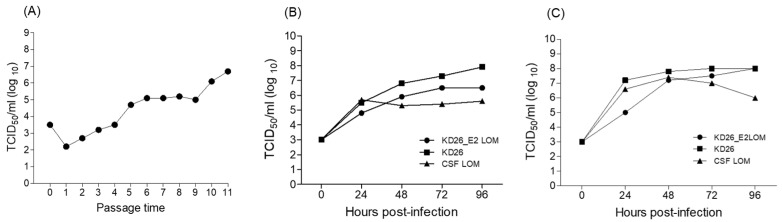
Proliferation of the KD26_E2LOM strain, the KD26 strain, and the CSFV LOM strain in cells. Titration of the KD26_E2LOM virus in PK-15 cells (**A**). Proliferation of the KD26_E2LOM strain, the KD26 strain, and the CSFV LOM strain in PK-15 (**B**) and MDBK (**C**) cells.

PK-15 cells transfected by electrophoresis of pACKD26_E2LOM showed low levels of infection with the KD26_E2 LOM strain after 24 h (Figure 2A); however, the infection spread to the surrounding cells at 36 and 48 h (Figure 2B,C). Fluorescence staining using a primary CSFV-E2 antibody revealed rings of specific fluorescence in the intracellular cytoplasm. In addition, to confirm that the E2 protein of the KD26_E2LOM virus was not derived from BVDV E2, but rather from the substituted CSFV E2, we used three specific monoclonal antibodies for the IFA analysis (Figure 2D–F). The KD26_E2LOM virus was subcultured in PK-15 cells for two generations and then detected by a CSFV E2-specific mAb (Figure 2D) and a BVDV Erns-specific mAb (Figure 2E); however, the KD26_E2LOM virus did not react with the BVDV E2-specific mAb (Figure 2F). The KD26_E2LOM strain, which was continuously passaged in PK-15 cells, showed the highest titer (10^6.7^ TCID_50_/mL) at the 11th passage (Figure 3A). In addition, inoculation of PK-15 cells with the KD26 strain resulted in high titers (10^7.9^ TCID_50_/mL) at 96 h, but the LOM strain yielded only low (10^5.7^ TCID_50_/mL) titers at this time (Figure 3B). Also, the KD26 strain showed a high titer (10^7.9^ TCID_50_/mL) at 96 h when inoculated into PK-15 cells, but the LOM strain showed a low titer (10^5.7^ TCID_50_/mL) (Figure 3B). However, inoculation of the KD26_E2LOM and KD26 strains into MDBK cells resulted in the highest titer (10^8.0^ TCID_50_/mL) at 96 h (Figure 3C).

### 3.3. Detection of Antibodies in Pigs Inoculated with KD26_E2LOM

**Figure 4:**
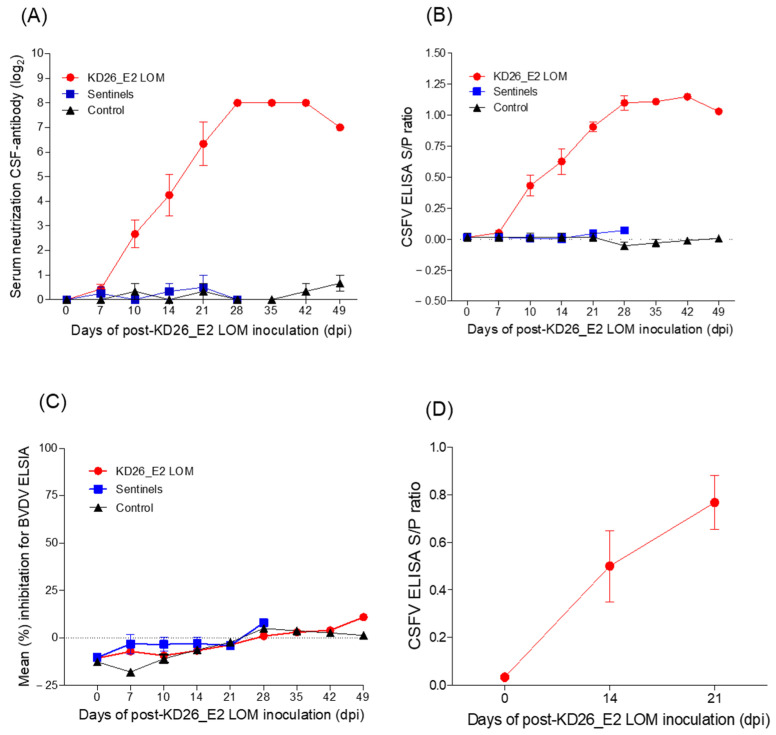
Antibody titers in growing pigs and pregnant sows inoculated with KD26_E2LOM. Serum-neutralizing anti-CSF-antibodies (**A**), the ELISA S/P ratio for CSFV E2-antibodies (**B**), and the ELISA for BVDV E2-antibodies (**C**) in growing pigs, and the ELISA S/P ratio for anti-CSFV E2 antibodies in pregnant sows (**D**). The KD26_E2LOM, sentinel, and control (mock) groups are marked by red circles, blue squares, and black triangles, respectively. Data are presented as mean ± standard deviation (SD).

Serum-neutralizing anti-CSF antibodies in growing pigs inoculated with KD26_E2LOM were seroconverted on Day 10 (average 2.67 log_2_), with the highest antibody (8.0 log_2_) titer maintained between Days 28 and 42 (Figure 4A). In addition, the CSFV E2-antibody ELISA S/P revealed seroconversion on Day 10 (average S/P ratio: 0.43) in the KD26_E2LOM-inoculated pigs, with the highest value (average S/P ratio: 1.15) determined on Day 42 (Figure 4B).

In growing pigs, the BVDV E2 antibody ELISA was negative for the KD26_E2LOM, KD26, and control groups until Day 49 (Figure 4C). When growing pigs were inoculated with KD26_E2LOM, they generated CSF-E2 and CSF-neutralizing antibodies, but not BVDV E2 antibodies, suggesting differential antibody functions. In addition, the average levels of serum-neutralizing anti-CSF antibodies detected in pregnant sows inoculated with KD26_E2LOM was 0.5 (log_2_) on Day 14 and 0.76 (log_2_) on Day 21, respectively (Figure 4D).

### 3.4. Shedding and Isolation of KD26_E2LOM from Growing Pigs and Pregnant Sows

After inoculation of growing pigs with a high dose (10^7.0^ TCID_50_/mL) of KD26_E2LOM, virus shedding was confirmed in one animal (in a nasal sample) at 7 dpi, and the virus was detected in blood samples taken on 1, 4, and 7 dpi (Table 3). We attempted to isolate the virus from PK-15 cells exposed to nasal and blood samples confirmed to be KD26_E2LOM-positive. The KD26_E2LOM virus was isolated from the nasal and blood samples from one animal (Table 3). However, no KD26_E2LOM was detected in the blood and nasal samples from pigs that co-inhabited with KD26_E2LOM-inoculated growing pigs, nor was it detected in the control pigs. In addition, although pregnant sows were inoculated with KD26_E2LOM at a high dose (10^7.0^ TCID_50_/mL) during the early stages (55–60 days) of pregnancy, we did not detect KD26_E2LOM RNA in the nasal or blood samples (Table 3).

### 3.5. Detection of KD26_E2LOM RNA in Organs from Growing Pigs and Pregnant Sows

Growing pigs inoculated with KD26_E2LOM were sacrificed and autopsied at 28 dpi KD26. Real-time qPCR was then conducted on tissue from 11 organs to detect E2LOM RNA. Viral RNA was detected in organs from five out of seven inoculated pigs; the virus was present mainly in tonsil, heart, kidney, spleen, cecum, and inguinal lymph nodes (Table 4). In addition, the KD26_E2LOM RNA copy number was high in tonsil (4.2 log_10_) and spleen (4.5 log_10_), and the KD26_E2LOM virus from these two organs was isolated successfully in PK-15 cells (Table 4).

Quantitative real-time PCR of organs from pregnant sows inoculated with KD26_E2LOM detected the virus in the cecum (Table 4). The RNA copy number in the cecum was 3.7 log_10_, and the virus was isolated from this sample when cultured with PK-15 cells (Table 4). The three pregnant sows (A8, A9, and A10) had 5, 10, and 14 fetuses, respectively, equating to 9.67 fetuses per sow (Table 5). The size of the fetal CR (cm) averaged 23.5 ± 0.5 (A8), 23.6 ± 1.9 (A9), and 21.2 ± 1.1 (A10) (Table 5). The qRT-PCR detected KD26_E2LOM virus in one fetus (3.7 log_10_) from sow A-9 (Table 5). In addition, the virus from the KD26_E2LOM-positive fetuses was isolated in PK-15 cells.

### 3.6. KD26_E2LOM and BVD26 Shedding in Calf

Five-day-old calves that did not consume colostrum were inoculated with KD26_E2LOM or BVD26, and viral shedding was investigated by qRT-PCR of blood, nasal, and fecal samples. At 2 dpi, the BVD26 virus was detected in fecal samples from 66.6% (2/3) of calves (Table 6). These fecal samples were inoculated into MDBK cells, and the BVD26 virus was isolated successfully. Also, the BVD26 virus was detected in the blood of one calf at 10 dpi, but the virus could not be isolated from the cell culture (Table 6). Viral antigen was detected in the blood of KD26_E2LOM-inoculated calves at 4 and 7 dpi (Table 6). In addition, the KD26_E2LOM antigen was detected in the nasal and fecal samples from one calf at 2 dpi, although the virus was isolated only from the fecal sample (Table 6).

## 4. Discussion

Since the early 1990s, researchers have tried to design new vaccines that can easily and accurately distinguish vaccinated individuals from those infected with CSF [27]. One of the most promising outcomes is a live attenuated DIVA vaccine, of which two vaccine types are representative: Chimeric *Pestivirus* Vaccines and Recombinant Deletion or Mutation DIVA Vaccines [27]. However, mutant DIVA vaccine strains generated by partial or complete deletion of genes via the introduction of artificial mutation sites have problems with respect to inducing immunogenicity and diagnostic methods for detecting DIVA [28,29,30,31]. By contrast, many studies of chimeric *pestivirus* have been conducted, and two vaccines have been licensed to date. The first is a chimeric *pestivirus* named “CP7_E2alf” (Suvaxyn^®^CSF Marker, Zoetis, Zaventem, Belgium), which was licensed as a DIVA vaccine in Europe in 2014. The second is Flc-LOM-BE^rns^, designated as a chimeric *pestivirus* and licensed in Korea in 2017. The chimeric *pestivirus* “CP7_E2alf” DIVA Vaccine is an infectious cDNA clone of the cytopathic BVDV strain “CP7”, in which the E2 gene was replaced by that of the CSFV variant “Alfort/187” [19,32,33]. The “CP7_E2alf” vaccine proved to be as safe and effective as a conventional live attenuating agent but was unable to prevent some cases of transmission through the placenta during pregnancy. It has therefore been argued that sows should not be vaccinated due to the risk of giving birth to PI offspring [34]. Another problem was the observed cross-reactivity between the “CP7_E2alf” vaccine and BDV- and BVDV-infected sera. Another chimeric *pestivirus* vaccine, licensed as Flc-LOM-BE^rns^, is based on the attenuated CSFV vaccine strain LOM [22]. This chimera was created by replacing the 30 bp terminal capsid gene and the complete Erns gene with the corresponding sequences from the KD26 BVDV strain. The Flc-LOM-BE^rns^ vaccine protected fetuses from transplacental infection with CSFV strain YC11WB during gestation (early, middle, or late). However, since this chimera vaccine was first used in Korea only in 2020, it is argued that it is difficult to evaluate its safety, or the robustness of the DIVA function, because there are insufficient data.

The KD26_E2LOM virus rescued in this study harbored mutations in four amino acid positions (257 in Npro, 1165 in E2, 1327 in NS2, and 2762 in NS4B) when compared with the original KD26 strain and the E2 gene of CSFV (LOM strain). The rescued KD26_E2LOM virus was detected specifically by an anti-CSFV E2-specific mAb and a BVDV E^rns^-specific mAb, which demonstrated DIVA function; however, it did not react with a BVDV E2-specific mAb. In addition, the proliferative capacity of the KD26_E2LOM virus differed according to cell type. MDBK cells contained the most KD26-E2LOM strain (average 10^8.0^ TCID_50_/mL) at 96 h. A *pestivirus* chimeric study revealed amino acid mutations in rC/bUTRs-tE2 after serial passage in PK15 cells: M834K and M979K in the E2 gene at passage 30 [21]. A comparison of the ability of the C-strain, rC/bUTR, and rC/bUTRs-tE2 to infect PK15 and MDBK cells revealed that all three viruses proliferated better in PK-15 cells than in MDBK cells; rC/bUTR showed the highest (10^4.5±0.3^ TCID_50_/mL) and rC/bUTRs-tE2 the lowest (10^3.1±0.2^ TCID_50_/mL) titers [21]. Another study demonstrated changes in seven amino acids (H450R and G479R in Erns, L686H in E1, and M783T, S973P, R1023H, and V1035E in E2) in the CP7_E2alf strain between the 1st and 11th passages [19]. After transfection of in vitro-transcribed CP7_E2alf RNA, autonomous replication of the chimeric RNA was observed in bovine and porcine cell cultures [19]. However, efficient growth of the chimeric CP7_E2alf virus could only be demonstrated in porcine cells, showing a similar growth pattern (>10^6.0^ TCID_50_/mL) to the Alfort 187 virus in PK15 cells [19].

Chimeric viruses “Ra”, “Pro”, and “RaPro” contain E^rns^ sequences from Norwegian mouse and Pronghorn *pestiviruses*, or combinations of both [23]. In pig cells, “Pro” chimeras replicate to high titers (10^5.3^ TCID_50_/mL), whereas “Ra” chimeras replicate less well (10^4.4^ TCID_50_/mL) [23]. Confirming the complete genome sequence of the three chimeras revealed several changes throughout the genomes of the “Ra” and “RaPro” chimeras, excluding “Pro” without mutation [23].

Here, we found that inoculating the target animal (i.e., pig) with KD26_E2LOM generated anti-CSF-E2 antibodies and CSF-neutralizing antibodies, but not anti-BVDV E2 antibodies, which is suggestive of DIVA function. Pigs inoculated with KD26_E2LOM generated anti-CSF E2 antibodies from Day 10, with the highest level maintained between Day 28 and Day 42. In addition, the ELISA S/P ratio for CSFV E2-antibodies in pregnant sows inoculated with KD26_E2LOM was positive from Day 14. After overdose-inoculation of KD26_E2LOM into growing pigs, we detected some viral shedding, and we isolated KD26_E2LOM from PK-15 cells. However, KD26_E2LOM RNA was not detected in the nasal cavity of sows inoculated with KD26_E2LOM during early pregnancy (Days 55–60). At 28 dpi, KD26_E2LOM RNA was detected in various organs harvested from five of seven pigs inoculated with KD26_E2LOM. The KD26_E2LOM virus was isolated from PK-15 cells cultured with positive samples.

A previous study developed an efficient marker, or DIVA, vaccine to help eradicate success in regions with a high prevalence of BVDV to prevent fetuses [24]. In the BVDV-1b_synCP7_DNpro_Erns Bungo vaccine, the viral protein E^rns^ was replaced with a heterologous E^rns^ from Bungowannah (BuPV, strain *pestivirus* F), and was attenuated by a deletion within the type I interferon inhibitor N^pro^ protein-encoding sequence [24]. The BVDV-1b_synCP7_DN^pro^_Erns Bungo_E1E2BVDV-2 CS vaccine, which is based on the BVDV-1b_synCP7_DNpro_E^rns^ Bungo vaccine, harbors the gene sequences of glycoproteins E1 and E2 from BVDV-2 strain CS8644 (CS) [24]. Both vaccines increased the titer to 10^6^ TCID_50_/mL, were safe for inoculation into cattle, and showed serological DIVA function [24]. A recent study developed chimeric viruses “Ra”, “Pro”, and “RaPro” that contain E^rns^ sequences from Norwegian mouse and Pronghorn *pestiviruses,* or a combination of the two [23]. All vaccine candidates were attenuated for use in swine vaccination/challenge trials, albeit to varying degrees. Serum samples from vaccinated animals have high neutralizing antibody levels that protect against virulent CSFV challenge and also did not cross-react in the CSFV E^rns^ antibody ELISA [23]. For a new generation of improved CSFV marker vaccines, E^rns^ antigens of distantly related *pestivirus* could act as a strong serological negative marker in chimeric *pestivirus* [23]. In another recent Japanese study, an infectious cDNA clone (DIVA) derived from CSFV live attenuated GPE-vaccine strains was used to convert glycoprotein E^rns^ of GPE-vaccine strains to E^rns^ of non-CSF *pestiviruses*, Pronghorn *pestiviruses* (PAPeV or Pocoena *pestiviruses*) [25]. High viral growth and genetic stability were confirmed in vitro after serial passage of the two chimeric viruses (vGPE-/PAPeV E^rns^ and GPE-/PhoPeV E^rns^) [25]. In vivo investigations also revealed that inoculation of vGPE-/PAPeV E^rns^ or vGPE-/PhoPeV E^rns^ at a dose of 10^4.0^ TCID_50_ resulted in immunogenicity and safety profiles comparable with those of vGPE-vaccine strains [25].

In the present study, among the three pregnant sows inoculated with the KD26_E2LOM strain, one was pregnant with five fetuses, which was lower than that of the other pregnant sows. However, no mummified fetuses were found in the pregnant sow with five fetuses, and the KD26_E2LOM strain was not detected in any of these five fetuses. Therefore, it seems likely that the reduced fetus count in this pregnant sow was due to low fertility that was not caused by inoculation with the KD26_E2LOM strain.

Virus from the caecum of pregnant sows inoculated with KD26_E2LOM was isolated from the PK-15 cell cultures, and the virus was also isolated from one fetus. This suggests a partial vertical infection by the KD26_E2LOM strain. We confirmed a partial placental passage of the KD2_E2LOM strain in a small-scale experiment performed within 3 weeks of mid-gestation. However, during the second trimester of pregnancy, if the fetus had been infected with the KD26_E2LOM strain for more than 3–4 weeks, the possibility of antibody formation would be expected to be higher than that of the formation of the KD26_E2LOM antigen. Although it is difficult to draw a clear conclusion due to the small number of experiments in the second trimester, we recognize that the developed KD2_E2LOM strain partially passed into the placenta. However, to address this issue fully, future experiments will need to be conducted with a larger number of pregnant sows in the early, middle, and late stages of pregnancy. When 5-day-old calves were inoculated with KD26_E2LOM, antigens were detected in the blood and feces, and the viruses were isolated from the fecal samples. This suggests the possibility of a horizontal infection when cattle are inoculated with the KD26_E2LOM strain. However, when high-titer KD26_E2LOM vaccine was inoculated into pigs and cattle, clinical symptoms similar to those in animals infected with CSFV or BVDV were not observed. Nevertheless, the safety of the KD26_E2LOM strain needs to be clearly confirmed through additional animal testing.

## 5. Conclusions

The KD26_E2LOM virus, which was created by inserting the CSFV LOM E2 gene into the BVDV backbone, is a DIVA vaccine capable of distinguishing between CSF E2 and BVD E2 antibodies. Pigs generated anti-CSF E2 antibodies from 10 dpi, with high antibody titers detected between 28 and 42 dpi. However, when inoculated with high doses, all calves and growing pigs shed the virus, which was isolated from positive samples using PK-15 cells. The KD26_E2LOM virus also suggested vertical infection from one pregnant sow to the fetus. Therefore, developed KD26_E2LOM shows DIVA function, but additional studies are required to assess its safety and ability to prevent infection by virulent CSFV.

## Figures and Tables

**Table 1:** Oligoprimers used to construct the KD26_E2LOM cDNA clone.

Primer		Sequence (5′-3′)	Location	Size
Construction of the plasmid for the full-length cDNA clone of KD26				
Frag 1	F	TTAAGCCCGGGC**TAATACGACTCACTATAG**TATACGAGAATTAGAAAAGG	1–21	695
	R	CCCGTCTATCACAGGGCCCCGCT	672–694	
Frag 2	F	TCACAGGGCCCCGCTGGAGCTCTTTG	680–705	1992
	R	GCACGAGAGAAACCAGATATCTCGCGATCTTGCA	2638–2671	
Frag 3	F	TACTGTGATGTCGATCGAAAGATTGGCTACATATGG	1887–1922	1701
	R	GGTCTTATCAGAACAGAAGGCCTCAGGGACT	3557–3587	
Frag 4	F	TATCAATTTAAAGAGAGCGAGGGACTACCACAC	3090–3122	2575
	R	GGATGGTCAGGATTGCCTATATTCGAAGCCT	5634–5664	
Frag 5	F	TATATTCGAAGCCTCCAGCGGGAGGG	5651–5676	1872
	R	CAGCACCTTTGTTTAAAGAAAACGTAGAAGCTGC	7489–7522	
Frag 6	F	GCAGGCTCAGCGTAGGGGCAGA	6650–6671	2396
	R	TGAAGAAGGGGTGTGCATTCACCTATGACC	9016–9045	
Frag 7	F	GGTGTGCATTCACCTATGACCTGACCATCTCC	9025–9056	1643
	R	CACCTGGTGGAACAATTGGTCAGGGATCTG	10,638–10,667	
Frag 8	F	AAAACACCTGGTGGAACAATTGGTCAGGG	10,634–10,662	1674
	R	GGACTAGGGAAGACCTCTAACAGCCCGGGCATCGAT	12,281–12,307	
Construction of the plasmid for the modified LOM-E2 plasmid				
LOM-E1	F	GCCGGCATTCTATGGCTGCTACTAGTAACTGG		
LOM-E2	R	CATAGTTTTAACAGAACAACCCGCCTCAGGGCC		

Note: The T7 promoter sequence is indicated in bold. Restriction enzyme sites are underlined. The sites are as follows: GCCCGGGC for *SrfI*, GGGCCC for *ApaI*, TCGCGA for *NruI*, CGATCG for *PvuI*, CCTNAGG for *Bsu36I*, TTTAAA for *DraI*, TTCGAA for *BstBI*, GCTNAGC for *BlpI*, GAATGCN for *BsmI*, CACNNNGTG for *DraIII*, and CCTNAGG for *Bsu36I*.

**Table 2:** Mutation of nucleotide and amino acid sequences in the KD26_E2LOM genome.

Position (Nucleotide Sequences)	Sequence Change		Related Gene
	Nucleotide (nt)	Amino Acid (aa)	
770	TCG → TTG	Ser → Leu	N^pro^
2423	CAG → CAA	Gln → Gln	E1
3495	TTG → GTG	Leu → Val	E2
3981	ATA → TTA	Ile → Leu	NS2
8286	ATG → GTG	Met → Val	NS4B

**Table 3:** Detection of viral RNA and isolation of KD26_E2LOM.

Group	Sample	Days Post-Inoculation (dpi)							
		0	1	4	7	11	14	21	28
G1	Blood	0/7 ^a^	1/7	1(+)/7	1/6	0/5	0/4	0/3	0/2
	Nasal	0/7	0/7	0/7	1(+)/6	0/5	0/4	0/3	0/2
G2	Blood	0/4	0/4	0/4	0/4	0/3	0/3	0/2	0/1
	Nasal	0/4	0/4	0/4	0/4	0/3	0/3	0/2	0/1
G3	Blood	0/4	0/4	0/4	0/4	0/4	0/4	0/4	0/4
	Nasal	0/4	0/4	0/4	0/4	0/4	0/4	0/4	0/4
G4	Blood	0/3	NT	NT	NT	NT	0/3	0/3	NT
	Nasal	0/3	0/3	0/3	0/3	NT	0/3	0/3	NT

G1: KD26_E2 LOM, 10^7.0^ TCID_50_/dose, single inoculation in growing pigs. G2: Growing pigs and pigs co-habiting with the growing pigs in G1. G3: Mock. G4: Pregnant sows (55–60 days) inoculated with 10^7.0^ TCID_50_/dose (KD26_E2 LOM). ^a^ Number of positive pig/total number of pigs. (+): virus isolated from cultured cells exposed to a positive sample. NT: not tested.

**Table 4:** RNA copy number in organs of pigs and sows inoculated with KD26_E2LOM.

Group	Pig	Dpi ^a^	RNA Copy Number (log_10_) in KD26-E2LOM Antigen-Positive Organs										
			To ^b^	He	Ki	Lu	Liv	Sp	Ce	Br	Il	Ln.1	Ln.2
G1	K1	4	-	-	2.9	-	-	-	-	-	-	3.6	-
	K2	7	3.7	-	-	-	-	4.5(+)	-	-	-	2.8	-
	K3	11	4.2(+)	-	-	-	-	-	-	-	-	-	-
	K4	14	-	-	-	-	-	-	3.1	-	-	-	-
	K5	21	-	-	-	-	-	-	-	-	-	-	-
	K6	28	-	3.5	-	-	-	-	-	-	-	3.9	-
	K7	49	-	-	-	-	-	-	-	-	-	-	-
G2	S1	7	-	-	-	-	-	-	-	-	-	-	-
	S2	14	-	-	-	-	-	-	-	-	-	-	-
	S3	21	-	-	-	-	-	-	-	-	-	-	-
	S4	28	-	-	-	-	-	-	-	-	-	-	-
G3	C1	49	-	-	-	-	-	-	-	-	-	-	-
	C2	49	-	-	-	-	-	-	-	-	-	-	-
	C3	49	-	-	-	-	-	-	-	-	-	-	-
	C4	49	-	-	-	-	-	-	-	-	-	-	-
G4	A8	21	-	-	-	-	-	-	-	-	3.7(+)	-	-
	A9	21	-	-	-	-	-	-	-	-	-	-	-
	A10	21	-	-	-	-	-	-	-	-	-	-	-

G1: KD26_E2 LOM, 10^7.0^ TCID_50_/dose, single inoculation in growing pigs. G2: Growing pigs and pigs co-habiting with the growing pigs in G1. G3: Mock. G4: Pregnant sows (55–60 days) inoculated with 10^7.0^ TCID_50_/dose (KD26_E2 LOM). ^a^ Dpi, days post-inoculation with KD26_E2LOM strain. ^b^ To, tonsil; He, heart; Ki, kidney; Lu, lung; Liv, liver; Sp, spleen; Ce, cecum; Br, brain; Il, ileum; Ln.1, inguinal lymph node; Ln.2, mesenteric lymph node. -: not detected. (+): virus isolated from cells cultured with positive samples.

**Table 5:** Crown-rump length and detection of KD26_E2LOM RNA in fetuses.

No. Pregnant Sow ^a^	Fetus	Crown-Rump Length (cm)													
		1	2	3	4	5	6	7	8	9	10	11	12	13	14
A-8	0/5 ^b^	23.5	24.0	23.0	24.0	23.0									
A-9	0/10	26.0	26.0	24.5	24.5	24.0(+) ^c^	23.5	22.5	23.5	22.5	19.5				
A-10	0/14	22.5	21.5	23.0	22.0	22.5	20.0	21.0	22.0	19.5	20.5	20.0	21.0	20.5	22.0

^a^ Pregnant sows (55–60 days) inoculated with 10^7.0^ TCID_50_/dose; ^b^ Number of dead fetuses/total births; ^c^ KD26_E2LOM-positive fetuses (qRT-PCR).

**Table 6:** Detection of viral antigen samples from calves inoculated with KD26_E2LOM or BVD26 strains.

Groups	Inoculation	No. Calf	Days Post-Inoculation (Blood/Nasal/Fecal) ^a^							
			−4	0	2	4	7	10	14	21
G1	KD26_E2LOM ^b^	3	0/0/0 ^a^	0/0/0	0/1/1(+)	1/0/0	1/0/0	0/0/0	0/0/0	0/0/0
G2	BVD26 ^b^	3	0/0/0	0/0/0	0/0/2(+)	0/0/0	0/0/0	1/0/0	0/0/0	0/0/0
G3	Mock	3	0/0/0	0/0/0	0/0/0	0/0/0	0/0/0	0/0/0	0/0/0	0/0/0

^a^ Number of blood/nasal/fecal-positive calves; ^b^ Calves receiving 10^7.0 ^TCID_50_/dose (5 days old); (+): virus isolated in MDBK cells.

## Data Availability

The data presented in this study are available from the corresponding author upon reasonable request.
